# A Painful Diffuse Rash

**DOI:** 10.1016/j.acepjo.2025.100290

**Published:** 2025-12-04

**Authors:** Bruce M. Lo, Melanie Shoemaker, Kiersten Potter, Katherine Schaffer

**Affiliations:** 1 Sentara Norfolk General Hospital/Eastern Virginia Medical School at Old Dominion University, Norfolk, Virginia, USA; 2 Eastern Virginia Medical School at Old Dominion University, Norfolk, Virginia, USA

**Keywords:** rash, monkeypox

## 1 Patient Presentation

**Figure 1:**
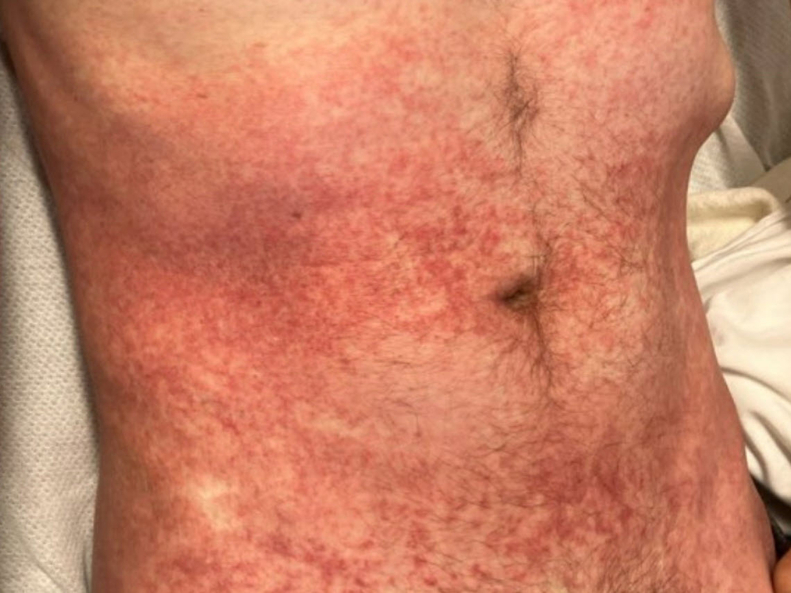
Macular papular rash to trunk.

**Figure 2:**
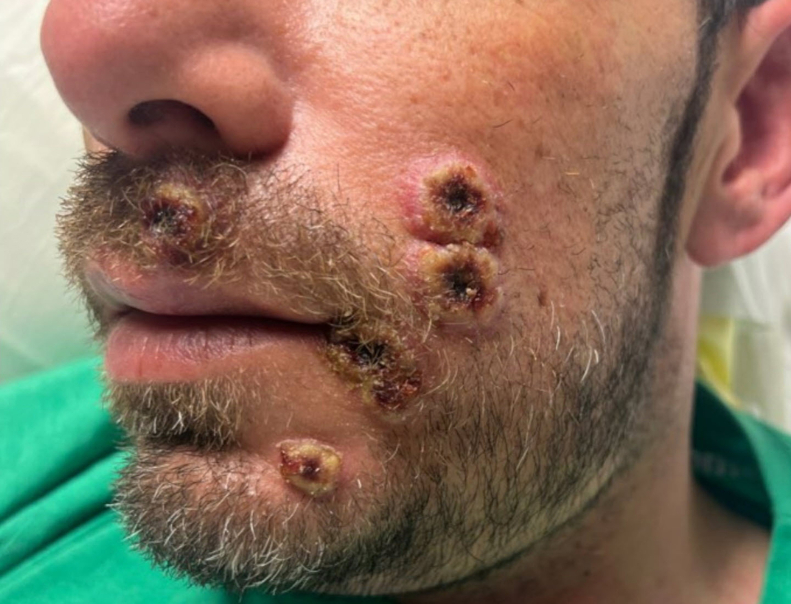
Facial ulcerations with central scabbing and rolled-up borders at the same stage.

A 41-year-old male with no past medical history presented with 1 week of ulcers to the left side of his face, followed by several days of a painful rash to the trunk and back after a sexual encounter. The patient denies any fever or previous rash and any illicit drug use. Initial vital signs included a temperature of 97 °F, a heart rate of 89 beats per minute, and blood pressure of 135/79 mm Hg. Physical examination revealed a macular papular rash across his trunk and back (Fig 1) and facial ulcerations with eschar (Fig 2) at the same stage. Laboratory findings showed a white blood cell count of 6.9 K/μL. Testing for human immunodeficiency virus (HIV), syphilis, herpes simplex virus, gonorrhea, and chlamydia was negative. Polymerase chain reaction (PCR) for monkeypox tested positive.

## 2 Diagnosis: Monkeypox

The incidence of monkeypox has surged, especially among men who have sex with men and immunocompromised populations. The typical presentation includes a prodrome of fever, lymphadenopathy, followed by a centrifugal, synchronous rash that evolves from macules to papules to pustules.^1^ The lesions tend to evolve at the same time on any given body part. Diagnosis of monkeypox is confirmed with PCR testing, and management is generally supportive, with tecovirimat reserved for severe or high-risk patients.^1^ Although incidence in the United States has remained low, emergency clinicians should consider monkeypox in the differential for high-risk individuals presenting with a diffuse rash, especially when the lesions are at the same stage on each part of the body.

## Funding and Support

By *JACEP Open* policy, all authors are required to disclose any and all commercial, financial, and other relationships in any way related to the subject of this article as per ICMJE conflict of interest guidelines (see www.icmje.org). The authors have stated that no such relationships exist.

## Conflict of Interest

All authors have affirmed they have no conflicts of interest to declare.

## References

[1] Al-Dabbagh J., Mohammad Deeb E., Younis R., Eissa R. (2024). The dermatological manifestations and differential diagnosis of monkeypox: a narrative review. Medicine (Baltimore).

